# Success Rate and Long-Term Effects of Embolization of Pelvic Arteries for the Treatment of Postpartum Hemorrhage

**DOI:** 10.1159/000527614

**Published:** 2023-02-21

**Authors:** Elisabeth Kapfhammer, Thomas Pfammatter, Romana Brun, Roland Zimmermann, Christian Haslinger

**Affiliations:** ^a^Department of Obstetrics, University Hospital Zurich, Zürich, Switzerland; ^b^Institute of Diagnostic and Interventional Radiology, University Hospital Zurich, Zürich, Switzerland

**Keywords:** Embolization, Postpartum hemorrhage

## Abstract

**Introduction:**

Postpartum hemorrhage (PPH) is the leading cause of peripartal maternal mortality and accounts for 25% of all maternal deaths worldwide. The most common reasons of PPH are uterine atony, retained placenta, or placenta accreta spectrum. Treatment of PPH depends on the etiology and corresponds to a stepwise approach, which follows the German, Austrian and Swiss guideline for the diagnosis and therapy of PPH in Switzerland. In severe ongoing PPH, hysterectomy has been the ultima ratio for many decades. Nowadays, interventional embolization of the pelvic arteries (PAE) has become a popular alternative. Besides being a highly effective minimally invasive method, PAE avoids hysterectomy with consecutively reduced morbidity and mortality. However, data on the long-term effects of PAE on fertility and menstrual cycle are scarce.

**Methods:**

We performed a monocentric study consisting of a retro- and a prospective part including all women who had undergone a PAE between 2012 and 2016 at University Hospital Zurich. Descriptive characteristics of patients and efficacy of PAE defined as cessation of bleeding were analyzed retrospectively. In the prospective part, all patients were contacted for a follow-up questionnaire regarding menstruation and fertility after embolization.

**Results:**

Twenty patients with PAE were evaluated. Our data showed a success rate of PAE in 95% of patients with PPH; only 1 patient needed a second, then successful, PAE. No patient needed a hysterectomy or any other surgical intervention. In our study, an association between mode of delivery and identified etiology of PPH is observed. After spontaneous delivery (*n* = 6), the main reason of severe PPH was retained placenta (*n* = 4), while after cesarean section (*n* = 14), uterine atony was identified in most cases (*n* = 8). Regarding menstruation after embolization, all women reported regular menstruation after the breastfeeding period (100%). The majority reported a regular pattern with a shorter or similar duration (73%) and lower or similar intensity (64%). Dysmenorrhea decreased in 67% of patients. Four patients planned another pregnancy, of whom only one had become pregnant with assisted reproductive technology and ended up in a miscarriage.

**Discussion:**

Our study confirms the efficacy of PAE in PPH, thus obviating complex surgical interventions and associated morbidity. The success of PAE does not depend on the primary cause of PPH. Our results may encourage the prompt decision to perform PAE in the management of severe PPH in case of failure of conservative management and help physicians in the post-interventional counseling regarding menstruation patterns and fertility.

## Introduction

According to the World Health Organization (WHO), postpartum hemorrhage (PPH) is the leading cause of maternal mortality and accounts for 25% of all maternal deaths worldwide [1, 2, 3]. Primary PPH complicates 4–6% of all pregnancies [4] and is a significant contributor to severe maternal morbidity and mortality [5]. The WHO defines PPH as blood loss greater than or equal to 500 mL within 24 h after birth, while severe PPH is blood loss greater than or equal to 1,000 mL within 24 h. The most common reasons for severe PPH are uterine atony (60%), followed by placental causes such as retained placenta tissue or placenta accreta spectrum (36%). Genital tract trauma (cervical and vaginal), uterine rupture, and maternal coagulopathies are less frequent but may also result in PPH [6]. Once hemorrhage is recognized, management needs to focus on achieving adequate uterine tone and maternal hemodynamic stability. In Switzerland, treatment of PPH follows the German, Austrian and Swiss guideline for the diagnosis and therapy of PPH (abbreviated DACH guideline), which recommends a stepwise approach [7]. First-line treatment includes administration of uterotonic agents, e.g., oxytocin or sulprostone and external uterine massage. Concurrently, laceration repair is indicated in cases of genital tract trauma and curettage in case of retained placenta. Managing a persistent bleeding despite uterotonic agents in cases of uterine atony may require the use of intrauterine balloon systems with or without vacuum [8, 9]. Surgical interventions like B-Lynch sutures, ligation of the uterine artery or the internal iliac artery as well as hysterectomy as ultima ratio have been the treatment for severe refractory PPH for many decades, especially peripartum hysterectomy is associated with high morbidity [10].

In 1979, Brown et al. [4] described the use of angiographic arterial embolization as a method to treat PPH and assumed the technique prior to surgical intervention [11]. According to the current DACH algorithm, embolization of the pelvic arteries (PAE) is suggested as a measure in case of persistent bleeding as a fourth step, in parallel to surgical interventions [7]. However, in our institution, obstetricians rather prefer to perform PAE instead of invasive surgical steps in case of failure of conservative management. The goal of uterine artery embolization (PAE) in the context of PPH is to occlude blood vessels temporarily with absorbable material to control bleeding [12]. PAE does not require general anesthesia and is not affected by coagulation disorder, which occurs regularly in case of ongoing severe bleeding [6]. The procedure has become popular as a highly effective method with success rates of 95% [13, 14] and might be initiated if administration of uterotonic drugs failed, the woman is hemodynamically stable, and the possibility of embolization is available [15]. Besides being minimally invasive, its main advantage is that it prevents hysterectomy [4, 5, 16] and preserves women's fertility. PAE does not seem to influence or impair menstruation according to surveys among women after PAE [17]. The aims of our study were, first, to evaluate retrospectively the efficacy of PAE as a measure for severe PPH irrespective of etiology of PPH or beforehand performed measures and, second, to analyze the long-term effects of PAE on menstruation and fertility in patients after PAE in our department.

## Methods

This single-center study was conducted at the Department of Obstetrics, University Hospital Zurich, Switzerland, in accordance with the guidelines for human participant research. Approval was obtained from the Local Ethics Committee (reference number KEK-ZH-Nr. 2016-00855). Inclusion criterion was the performance of PAE due to severe PPH, irrespective of time point of PAE or etiology of PPH, in the study period between January 2012 and December 2016. At our institution, we follow the WHO definition of PPH; i.e., severe PPH was diagnosed in case of measured blood loss exceeding 1,000 mL and secondary PPH was diagnosed in case of hemorrhage between 24 h and 12 weeks postpartum. Retained placenta was defined as duration of third stage of labor of more than 30 min. The deliveries took place at the Department of Obstetrics and the PAE at the Department of Interventional Radiology in the same building. This study contains a retro- and a prospective part.

For the retrospective part, we analyzed data collected between 2012 and 2016. The cases were analyzed regarding patient characteristics and clinical course during PPH, leading to the decision to perform PAE. The characteristics concerning maternal variables were collected from our electronic database “Perinat” (a University Hospital Zurich product) and contained maternal age, parity, history of PPH, type of gestation (singleton or multiple), and type of delivery. The following parameters concerning PPH were extracted from the electronic database as far as possible: cause of PPH, interventions performed before embolization, and medication administered during PPH treatment. Outcome parameters included the success of PAE in stopping bleeding, the amount of blood transfusion, and duration of hospitalization.

Blood loss was analyzed by measuring blood loss and ∆hemoglobin (Hb, difference between prepartum Hb and last Hb before PAE). For vaginal deliveries, our institution has been using a validated quantitative system to measure blood loss, which was shown to have an excellent correlation between measured and calculated blood loss.

In the prospective part of this study, all patients treated with PAE were contacted to obtain information about menstruation patterns and fertility after embolization. Follow-up time ranged from 4 weeks to 4 years after the delivery and PAE. The patients were asked to answer a questionnaire including the following questions.

• Has your menstruation already started after the delivery and following intervention?

• Is your menstruation in a regular manner as you were used to (before pregnancy)?

• Has the intensity of your menstruation changed after embolization?

• Has the intensity of pain during your menstruation changed compared to the situation before the delivery and following intervention?

• Have you tried to get pregnant again?

• If yes, have you had another delivery or any miscarriage since the delivery and PAE?

Exclusion criterion for the questionnaire was an amenorrhoic status due to breast feeding after delivery.

### Technique of PAE Performed at Our Institution

**Fig. 1:**
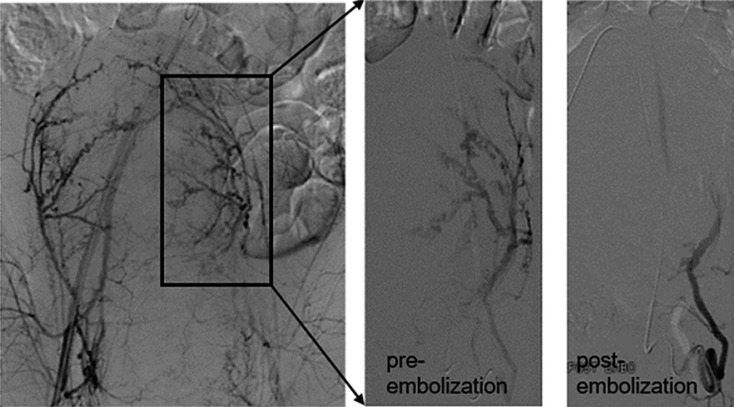
PAE pre- and post-embolization.

The interventions were performed in a dedicated angiography suite. After applying local anesthesia, unilateral percutaneous femoral artery access was gained. In selected cases, the pre-installed epidural or general anesthesia was maintained throughout the embolizations. 4 F catheters (Cobra C2, Cordis Corp., Miami Lakes, FL, USA) were advanced into both uterine arteries or at least into the anterior divisions of the respective internal iliac arteries. In some cases, at the discretion of the interventional radiologist, coaxial microcatheters (Progreat®, 2.7 F, Terumo Corp., Leuven, Belgium, China) were utilized to engage the uterine arteries. Depending on the chosen catheter, embolizations were performed either with gelfoam pledgets or gelfoam slurry, which are considered temporary embolic agents, until flow stasis in the uterine arteries was achieved (Fig. 1).

## Results

Twenty patients who underwent PAE due to severe PPH at the Department of Obstetrics at the University Hospital Zurich between January 2012 and December 2016 were included. PAE was successful in 19 of 20 patients (95%) after one intervention. In the unsuccessful case, hemorrhage restarted 48 h after PAE and continued despite administration of oxytocin and sulprostone. The patient needed a transfusion of 2 units of RBC which did not result in an adequate increase in Hb levels. Thus, a second PAE was performed successfully 72 h after the first attempt. None of our patients needed a hysterectomy or any other surgical intervention after PAE. Baseline characteristics of the study population as well as etiology of PPH are provided in Table 1.

Severe PPH occurred in 30% (*n* = 6) after vaginal and in 70% (*n* = 14) after cesarean section (among these, 21% were elective and 79% unplanned cesareans). Five women had a cesarean section due to twin pregnancies, four of them were preterm deliveries.

In our study, the etiology of PPH was different between cesarean and vaginal deliveries. After spontaneous delivery (*n* = 6), PPH occurred in 66.7% (*n* = 4) due to retained placenta and in 33.3% (*n* = 2) due to uterine atony. After cesarean section (*n* = 14), the main cause of PPH was uterine atony (57.1%, *n* = 8); in 14.3% (*n* = 2), PPH was caused by placenta accreta spectrum; and only one case of PPH (7.1%) was caused by coagulopathy as part of HELLP syndrome (HELLP: hemolysis, elevated liver enzyme levels, and low platelet count). In the remaining three cases, PAE was performed due to secondary PPH due to uterine atony, in our study population eight, respectively, 9 days after delivery.

Conservative management before PAE was performed as follows: 100% received oxytocin, 95% received misoprostol, 75% sulprostone, and 20% intrauterine balloon tamponade (Bakri® balloon). Coagulation factors (fibrinogen, factor XIII) were administered to 65% of women. Six patients with uterine atony and suspected retained placenta tissue had a curettage without success in cessation of bleeding.

The measured blood loss before initiation of PAE differed by the mode of delivery. After spontaneous delivery, the median measured blood loss was 3,500 mL (range 2,000–5,700 mL); after cesarean section, the blood loss was 1,600 mL (range 1,000–2,500 mL). Erythrocytes were administered to 30% of the patients with a median of four packed red blood cells; in one case, the transfusion of platelets was necessary. Coagulation factors (fibrinogen and coagulation factor XIII) were administered in 65% of cases and tranexamic acid in all cases. The median decrease in the Hb level between hospital admission and immediately before embolization was 44.1 g/L (range 5–87 g/L). All patients were monitored in the ICU for a maximum of 24 h after PAE.

The duration of hospitalization differed according to mode of delivery. After a spontaneous delivery, patients stayed 3–4 days; after a caesarean section, length of stay was 5–6 days.

**Fig. 2:**
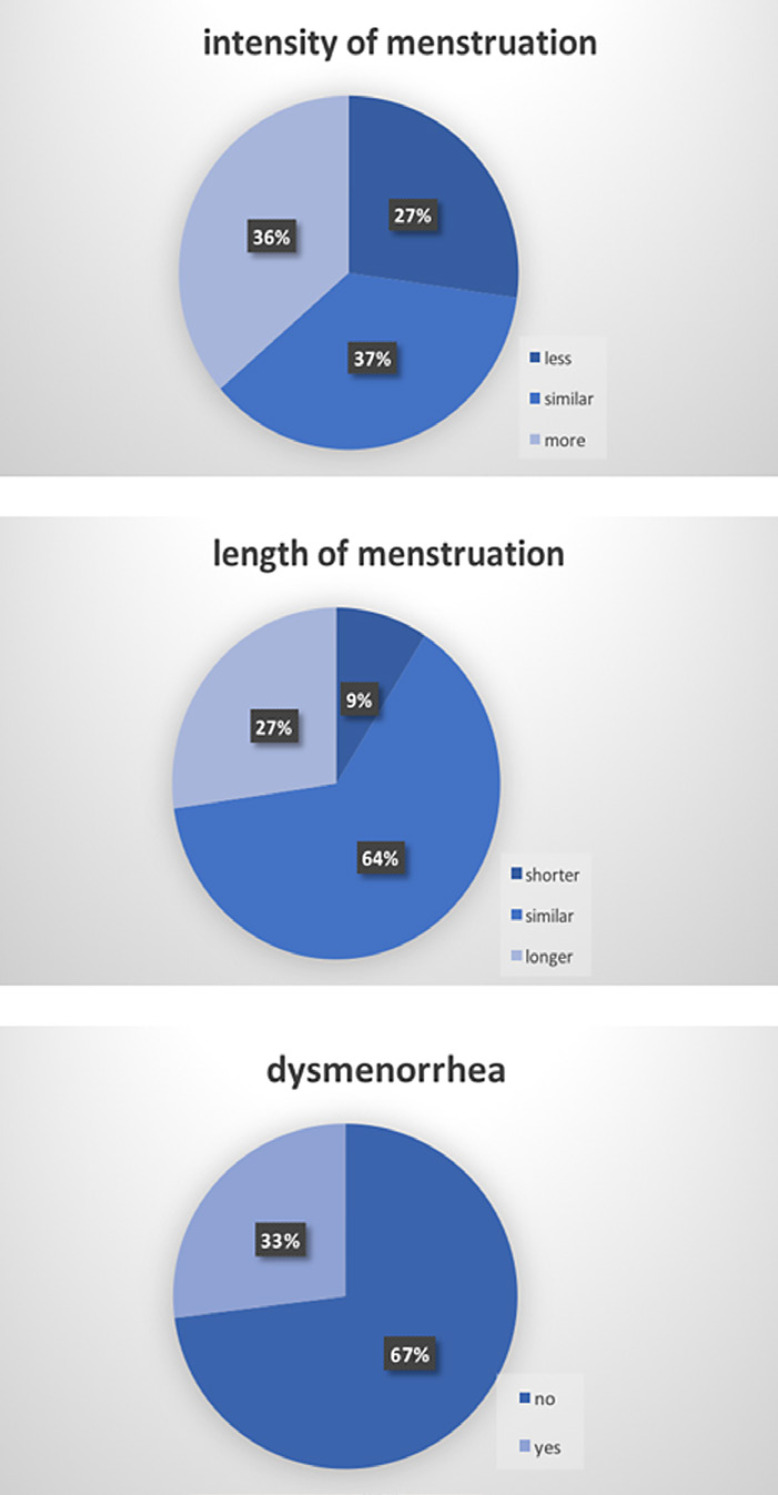
Menstruation pattern after PAE.

With reference to the prospective part of our study, twelve women completely answered the survey about menstruation pattern and fertility after PAE (60%). All women reported regular menstruation after they had stopped breastfeeding (Fig. 2).

The majority of the patients (64%) reported a lower or similar intensity of their menstruation compared to the situation before embolization; 36% complained about hypermenorrhea after PAE. Likewise, a majority of 73% had a shorter or similar duration of menstruation than before. Furthermore, dysmenorrhea was decreased in 66.6% of patients after PAE.

Among the patients who responded to the follow-up, only four planned to get pregnant again after embolization. Two of them had already had an assisted reproduction procedure in their history and were planning another in vitro fertilization for the next pregnancy. So far, only 1 patient got pregnant, 3 years after PAE. This pregnancy resulted from intrauterine insemination and ended in a miscarriage.

## Discussion

Our study confirms the effectiveness of PAE in women with severe PPH. PAE was successful in 19 out of 20 women (95%) after one PAE; only 1 patient had a recurrence of hemorrhage after several days, which was stopped after a second PAE. No patient needed surgical measures via (re-)laparotomy or had postpartum hysterectomy. Success of PAE did not depend on the primary cause of PPH, and no major complications due to PAE were observed. This finding goes along with the existing literature, which states major and serious complications like dissection of the uterine arteries, transient numbness of the lower extremities, or hematoma at the puncture site to be rare after PAE [12].

However, the availability of PAE is limited to centers with a service of interventional radiology 24 h a day, 7 days a week. Therefore, this treatment modality is not available to all women with severe PPH. This reinforces the importance of planned delivery or transfer of women with high risk for severe PPH to adequately equipped centers, as recommended by the DACH guideline. According to the Royal College of Obstetrics and Gynaecology guideline, women with suspected placental abruption (OR, 13), known placenta previa (OR, 12), multiple pregnancy (OR, 4), or preeclampsia/gestational hypertension (OR, 4) are regarded as highest risk for PPH [18].

Dahlke et al. [19] performed a descriptive analysis of four national guidelines (Royal College of Obstetrics and Gynaecology, American College of Obstetricians and Gynecologists, Royal Australian and New Zealand College of Obstetricians and Gynaecologists, and Society of Obstetricians and Gynaecologists of Canada) regarding prevention and management of PPH. All guidelines discussed the role of surgical techniques as well as PAE and recommended promoting less invasive fertility-sparing interventions [19], which is consistent with a retrospective analysis suggesting that PAE should be considered early in management of PPH [12]. The authors state that even hemodynamic instability should not be considered a contraindication for PAE and repeat embolization should be done before performing hysterectomy due to its advantage in achieving hemostasis without sacrificing the reproductive ability of the woman. The significance of the latter statement is underlined by the fact that, in our collective, 65% of women had PAE due to severe PPH after their first delivery.

Besides preservation of fertility, PAE avoids more complex surgical interventions and prevents women from postpartum emergency hysterectomy, which is associated with a high risk for mortality or severe morbidity such as massive blood loss and postprocedural maternal morbidity including cardiac arrest, DIC, pulmonary edema, pneumonia, and bladder injury [20, 21]. A further advantage of successful PAE is improved convalescence and shorter duration of hospitalization as compared to complex surgical interventions such as postpartum hysterectomy [21]. This finding was also observed in our collective as women left hospital after 3–4 days (after vaginal deliveries) and 5–6 days (after cesarean sections) despite significant blood loss. The abovementioned advantages of PAE lead to the recommendation to perform PAE even in hemodynamic instability and DIC, which are known to be poor predictors in PPH, presupposed a close monitoring during and observation after PAE [12].

Regarding effects on menstruation and fertility after PAE, our results are consistent with current studies, which claim no harm to the endometrium through PAE [17, 22, 23, 24]. Also, in our study, all women resumed a regular menstruation after the breastfeeding period. The majority of our patients reported a reduced duration and intensity of menstrual bleeding and less dysmenorrhea. Although not being asked about this issue, the majority of women reported on the questionnaire spontaneously about their satisfaction with the received treatment, especially in the light of having kept their uterus and, consequently, their fertility.

Concerning fertility after PAE, the scarce number of women in our study who actually attempted to become pregnant again does not allow any conclusive findings. However, different studies expect to have a return of normal menses with preservation of future fertility and successful pregnancies [17, 18, 24, 25]. In our study, we gained information about the long-term effect of PAE on menstruation patterns, which we consider a strength of this study. Based on the current body of evidence, the return of a regular menstruation allows to assume preserved fertility.

Our study is limited by its low number of cases in general, owing to the rare incidence of refractory PPH in our institution. Another limitation is the retrospective character of the analysis which does not allow a detailed description of conservative treatment steps before performance of PAE. Furthermore, we acknowledge that at our institution, surgical steps such as compression sutures are used markedly seldom in contrast to comparable centers. Consequently, the aim of this study was to assess the efficacy and long-term effects of PAE and not a comparison with other measures. Due to the low case number, long-term effects of PAE regarding fertility are difficult to assess in a single-center study. Another limitation is a long time between intervention and follow-up in some cases, which might impede the women's subjective answers to the questionnaire. On the other hand, the long time after intervention can also be seen as an advantage, as a longer follow-up regarding menstruation pattern is possible.

In conclusion, at our institution, PAE had a high success rate in women with severe PPH (95% at first attempt), independent of the etiology of PPH with no need for additional surgery and no observed intervention-related adverse outcome. The analysis of menstruation pattern was reassuring as all women had regular menses after cessation of breastfeeding, a finding which might help physicians during post-interventional counseling of the patients. Thus, PAE remains an important tool for the treatment of severe PPH after failure of conservative management before surgical measures become necessary.

## Statement of Ethics

Our research complies with the guidelines for human studies and includes evidence that the research was conducted ethically in accordance with the World Medical Association Declaration of Helsinki. Our patients have given their written informed consent. Approval was obtained from the Local Ethics Committee, Zurich (reference number KEK-ZH-Nr. 2016-00855).

## Conflict of Interest Statement

The authors do not report any potential conflicts of interest. The manuscript has been seen and approved by all authors, and we affirm that the manuscript has not been published previously and is not being considered concurrently by another publication.

## Author Contributions

E. Kapfhammer performed the research and wrote the article. C. Haslinger, R. Brun provided intellectual input. Th. Pfammatter provided radiological input and R. Zimmermann reviewed the manuscript.

## Data Availability Statement

All data generated or analyzed during this study are included in this article. Further inquiries can be directed to the corresponding author.

## Figures and Tables

**Table 1:** Baseline characteristics of the study population and etiology of PPH

	All patients with PAE (*n* = 20)	Subgroups according to delivery mode	
		PAE after cesarean section (*n* = 14)	PAE after spontaneous delivery (*n* = 6)
Baseline characteristics, *n* (%)			
Age, years	35.5 (32.3–40)	37 (34.3–40)	32.5 (28.5–35)
Primiparity	11 (65)	8 (57.1)	3 (50)
Multiparity	9 (35)	6 (42.9)	3 (50)
Twin pregnancy	5 (25)	5 (35.7)	0
History of PPH	2 (10)	1 (7.1)	1 (16.67)
Etiology of PPH, *n* (%)			
Atony	10 (50)	8 (57.1)	2 (33.3)
Placenta accreta spectrum	6 (30)	2 (14.3)	4 (66.7)
HELLP syndrome	1 (5)	1 (7.1)	0
Secondary PPH	3 (15)	3 (21.4)	0
Outcome			
Measured blood loss, mL	2000 (1350–2500)	1600 (1000–2000)	3,500 (2550–4,975)
Delta Hb, g/L	44 (26–64.5)	32 (23–58.5)	64 (62–68)
Need for blood transfusion, *n* (%)	7 (35)	3 (21.4)	4 (66.7)
